# Effect of different irrigations on the bond strength of self-adhesive resin cement to root dentin

**DOI:** 10.4317/jced.54459

**Published:** 2018-02-01

**Authors:** Carlos Garcia, Edwin Ruales-Carrera, Luiz-Henrique-Maykot Prates, Claudia-Angela-Maziero Volpato

**Affiliations:** 1 DDS, MSc, Department of Dentistry, Federal University of Santa Catarina (UFSC), Florianópolis, SC, Brazil; 2 DDS, MSc, PhD Student, Center for Education and Research on Dental Implants (CEPID), Department of Dentistry, Federal University of Santa Catarina (UFSC), Florianópolis, SC, Brazil; 3 DDS, MSc, PhD, Department of Dentistry, Federal University of Santa Catarina (UFSC), Florianópolis, SC, Brazil

## Abstract

**Background:**

Different disinfectant solutions or medications are indicated prior fiber post bonding procedures.The aim was to evaluate the effect of different pretreatments of root canal on the bond strength of a fiber post luted to dentin with self-adhesive resin cement.

**Material and Methods:**

Forty-eight single-rooted mandibular premolars were endodontically treated and prepared to receive fiber posts. Teeth were assigned to four groups (n=12). Root canal walls were subjected to no pretreatment (G1 - control); irrigation with 10 mL of 5% sodium hypochlorite (NaOCl) (G2); 10 mL of 17% ethylenediaminetetraacetic acid (EDTA) associated with 10 mL of 5% NaOCl (G3); or 10 mL of 17% EDTA (G4). Fiber posts were bonded with self-adhesive cement. After the roots were sectioned into slices, a push-out bond strength test was performed using a universal testing machine at crosshead speed of 0.5 mm/min. Bond strength data were recorded and expressed in MPa and analyzed by Anova (5%) and Tukey Test (5%).

**Results:**

It was found that G2 (9.36 MPa) and G4 (6.33 MPa) were significantly different among themselves and statistically inferior to G1 (13.93 MPa) while G3 (14.31 MPa) was statistically similar to G1 (control) and superior to G2 and G4.

**Conclusions:**

Irrigation with 17% EDTA associated with 5% NaOCl showed increased bond strength compared with the same solutions used alone.

** Key words:**Self-adhesive, Dentin, Push-out, Bonding.

## Introduction

Posts and cores are commonly used in endodontically treated teeth suffering from excessive coronal tooth structure loss (1-3). The selection of an appropriate restoration method for endodontically treated teeth is guided by both strength and aesthetic results (4). Fiber posts are currently widely used in combination with resin cements to restore tooth function (1,2,5,6). In addition, the elastic modulus of fiber posts, similar to that of dentin, results in homogeneous stress distribution along the root canal. As a result, fiber posts minimize the risk of root fracture or lead to a more favorable fracture mode than metallic posts (7,8).

Fiber posts have additional advantages, such as biocompatibility, corrosion resistance, satisfactory aesthetics, improved light transmission, chemical compatibility with adhesive systems and resin cements, placement in a single visit waiving indirect procedures and easier removal compared to metallic posts (2,4,5).

Resin cements are widely used for cementing fiber due to their compatibility to adhesive systems. Self-adhesive cements have brought clinical benefits to overcome some limitations of conventional and self-etch resin cements. The system does not require any pretreatment to remove the smear layer from the root canal, and once the cement is mixed the application is done in a single clinical step (3). Self-adhesive cements simultaneously demineralize and infiltrate enamel and dentin because of multifunctional monomers with phosphoric acid groups (9). It is claimed that the adhesion obtained relies on micromechanical retention and chemical interaction between monomer acidic groups and hydroxyapatite (10-12).

It is suggested that the use of some disinfectant solutions or medications during root canal preparation may have an adverse effect on the bond strength between fiber post and root dentin (13-15). Sodium hypochlorite (NaOCl), the most commonly used irrigant, is considered an excellent proteolytic agent and leads to greater dissolution of organic compounds (mainly collagen) in dentin during root canal therapy. However, it does not completely remove the smear layer (16,17).

Ethylenediaminetetraacetic acid (EDTA) is another agent indicated for smear layer removal, as it reacts with calcium ions in dentin and forms soluble calcium chelates (16). A literature review by Violich & Chandler (18) included some classic studies that suggested the alternate use of EDTA and NaOCl would be appropriate for smear layer removal due to their intrinsic properties.

Given the different possibilities of final root canal irrigation prior to fiber post cementation with self-adhesive resin cements that do not require smear layer removal, this study aims to clarify whether different irrigation solutions influence bond strength. The study results can contribute to establish a protocol for fiber post cimentation. This study used the push-out test to evaluate the bond strength of fiber posts luted with self-adhesive resin cement to root canal dentin under different disinfection techniques. Single or combination solutions of 17% EDTA and 5% NaOCl were used during the final irrigation procedure after root canal preparation and prior to fiber post cementation.

The null hypothesis was that the irrigant solutions used at the end of the root canal preparation phase and prior to the cementation procedure with self-adhesive resin cement do not interfere in the bond strength to the root dentin.

## Material and Methods

Approval to use 48 extracted human mandibular premolars with single canals was obtained from the Federal University of Santa Catarina ethics committee (no. 120113) for this study. The inclusion criteria were absence of caries or root cracks and absence of previous endodontic treatments, posts, or crowns. A slow-speed diamond saw was used to decoronate the teeth 1 mm coronally to the cement-enamel junction to obtain standardized root remnants of 16 mm ± 1 mm (Isomet, Buehler Ltd., Lake Bluff, Ill, USA). The same operator performed manual endodontic treatment of the root remnants using the apex-crown regressive technique with stainless steel K-Files. Canal working lengths were established 1 mm shorter to the apical foraminal openings and the scaling was finished with a #60 file (Dentsply Maillefer, Ballaigues, Switzerland). During instrumentation, the canals were irrigated with 2 mL of 1% NaOCl (Cloro Rio, Rioquímica, São José do Rio Preto/SP, Brazil).

To remove the smear layer, 3 mL of 17% trisodium EDTA (Titriplex III PA, Merck, Germany) pH 7.5 solution was used, followed by 3 mL of 1% NaOCl (Cloro Rio, Rioquímica, São José do Rio Preto/SP, Brazil) solution. After mechanical and chemical preparation, the root canals were rinsed with 10 mL distilled water, dried with paper points (Tanari, Manacapuru/AM, Brazil), and filled with gutta-percha cones (Tanari, Manacapuru/AM, Brazil) and calcium hydroxide and non-eugenol sealer (Sealer 26; Dentsply-Maillefer, Petrópolis/RJ, Brazil).

Following endodontic treatment completion, cervical root canal openings were filled with a provisional restorative material (Cavitec; Caithec Industrial, Rio do Sul/SC, Brazil) and stored at 37°C and 100% humidity (distilled water) for seven days. Post spaces were prepared in depths of 10 - 12 mm with a low-speed drill provided by the post system manufacturer (White Post – DC 2; FGM-Dentscare, Joinville/SC, Brazil), maintaining an apical seal of approximately 4 mm. Following post space preparations, the 48 roots were randomly divided into four groups (n=12) and irrigated, as shown in  Table 1.

**Table 1:**
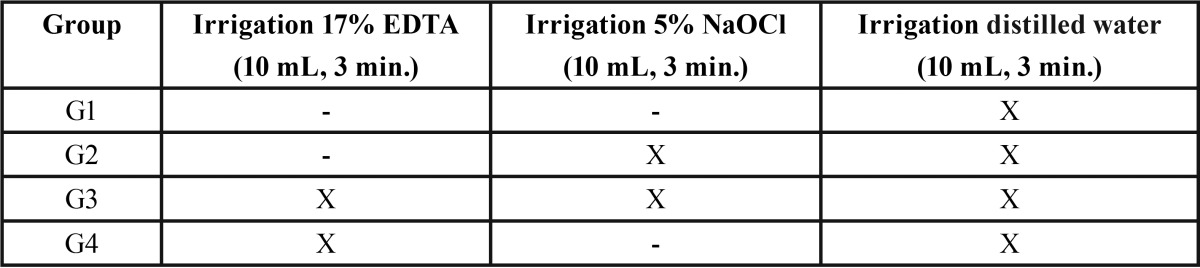
Sequence of fiber post pre-cementation irrigation in each group.

**Figure 1:**
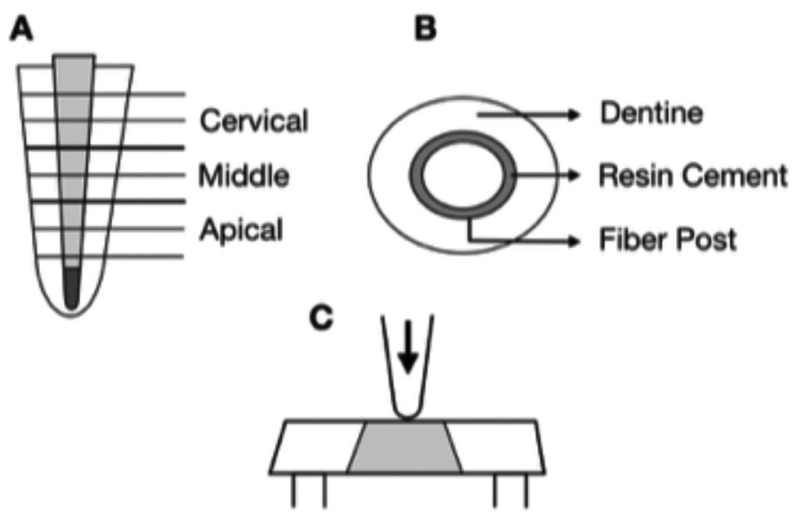
(A and B) Schematic view of specimen preparation for push-out test. Six segments of 1 ± 0.1 mm thickness were obtained, two for each third (cervical, middle and apical). (C) Sliced specimen prepared for push-out test.

Before cementation, each fiber post was cleaned with 70% alcohol and dried; a silane agent was applied according to manufacturer’s instructions (Prosil; FGM-Dentscare, Joinville/SC, Brazil). For fiber post cementation, equal amounts of self-adhesive resin cement luting paste (RelyX U100; 3M ESPE, Seefeld, Germany) were hand mixed and applied on post surfaces and inside root canals using a Weston probe (Golgram, São Caetano do Sul/SP, Brazil). Subsequently, fiber posts were placed inside the root canals and kept under finger pressure until the end of the cementation process; the cement excess was removed with a spatula. The specimens were polymerized with the tip of a LED light unit (Ultralume - Ultradent, USA) in direct contact with the coronal end of the posts for 40 s, at 600 mW/cm2 intensity, measured with a radiometer (Kerr Demetron - West Collins Orange, CA, USA). Finally, the teeth were identified and stored at 37°C and 100% absolute humidity (distilled water) for 24 h until complete cement polymerization. Twenty-four hours after fiber post cementation, the specimens were positioned and cut perpendicular to the long axis using a diamond saw (Isomet, Buehler Ltd., Lake Bluff, IL, USA), under water cooling. The first slice, corresponding to the coronal portion, was discarded. Six segments (1 ± 0.1 mm thickness) were obtained, two for each third (cervical, middle and apical) as shown in Figure 1A,B).

-Push-out test 

The push-out test better simulates the clinical conditions and is more appropriate, reliable and reproducible than the micro tensile test (19-21). It was thus selected to evaluate the bond strength in this study.

The push-out test was performed using a universal testing machine (Instron 4444, Canton, MA, USA) at crosshead speed of 0.5 mm/min, with two pins (diameters 0.6 mm and 1 mm) in the center of the apical surface of the post in an apical-coronal way, without stressing the surrounding post space walls, until bond failure occurred (Fig. 1 C).

To express the bond strength in megapascals (MPa), the force required to displace the specimen in newtons (N) was divided by the area (mm2) of the adhesive dentin/post interface. The following formula was applied to calculate the area of adhesion: A = π. g. (r1 + r2), which corresponds to the lateral area of a truncated cone, observed in the post, where π is the constant 3.14; g = generatrix of truncated cone; r1 = smaller base radius; r2 = larger base radius, h = section height. The Pythagorean theorem was used to calculate the generatrix (g), i.e. (g2 = h2 + [r2-r1] 2). The thickness of each slice was measured using a digital caliper to record the fiber post thickness (height, h). A measuring microscope (Mitutoio, Japan) with 1500x magnification and 0.005 mm accuracy was used to measure the diameter of the largest and smallest circumference of each section.

Two specimens of each group were analyzed with a SEM after the push-out test was performed, for illustrative purposes.

-Statistical Analysis 

Residual distribution normality was initially assessed by the Kolmogorov Smirnov test. A value of p: 0.167 was obtained which indicated sample normality and allowed the use of Anova and Tukey tests (5%) to verify similarities and significant differences between groups.

## Results

**Figure 2:**
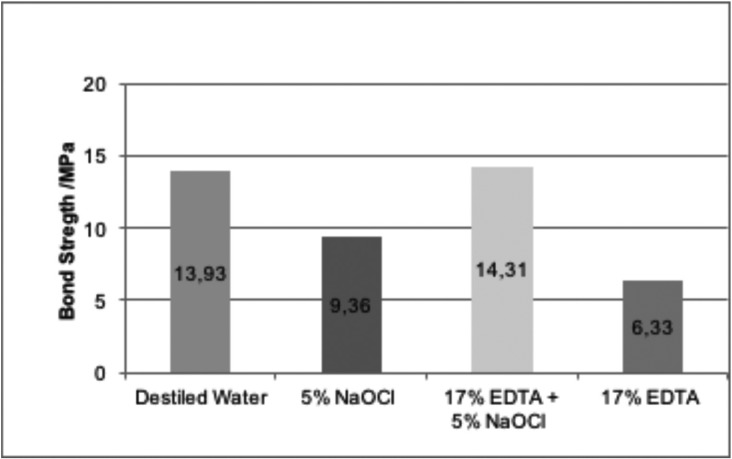
Mean bond strength values (MPa) and respective groups.

The means and standard deviations of push-out bond strengths are described in  Table 2 and illustrated in Figure 2. The following similarities and differences were obtained based on the statistical analysis using Anova and Tukey tests (*p*=0.05): G2 (5% NaOCl) - 9.36 MPa and G4 (17% EDTA) - 6.33 MPa, provided average bond strength values significantly different among themselves (*p* = 0.013027), both statistically inferior to G1 - 13.93 MPa (control) (G1 X G2, *p* = 0.000255 and G1 X G4, *p* = 0.000169). On the other hand, when both irrigating solutions were combined in G3, the bond strength of 14.31 MPa was statistically similar to G1 (control) (*p* = 0.977994) and greater than G2 (*p* = 0.000188) and G4 (*p* = 0.000169).

**Table 2:**
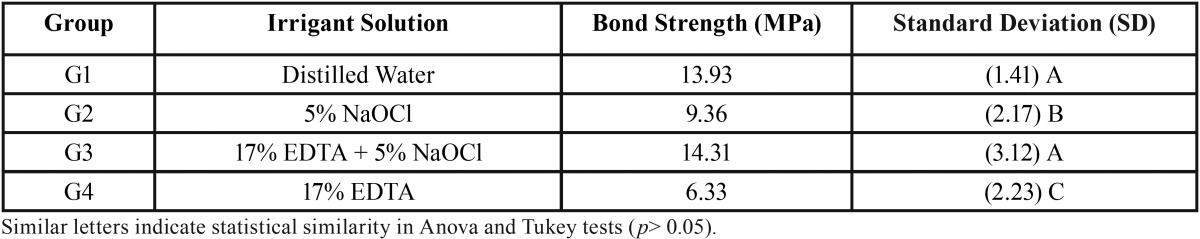
Mean bond strength values (MPa) and respective standard deviations for the groups evaluated.

**Figure 3:**
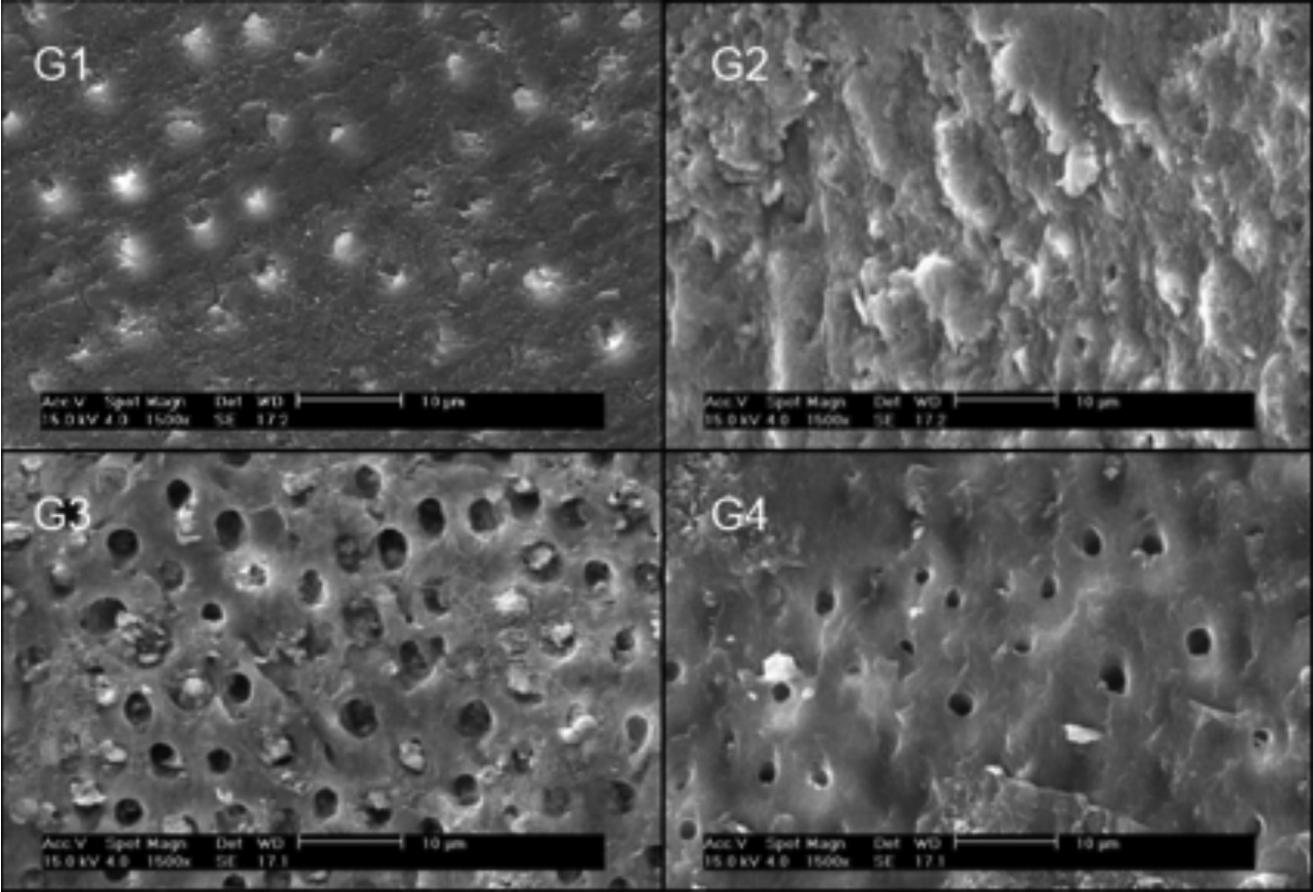
Scanning electron micrograph (1500x) showing characteristics of each group’s root dentin surface after the push-out test. G1 (Control –distilled water): dentin surface showing tags resin or smear plug (arrow). G2 (5% NaOCl): dense smear layer and resin cement on dentinal surface occluding tubules. G3 (17% EDTA + 5% NaOCl): dentin surface after smear layer removal, with open dentinal tubules and some resin tags. G4 (17% EDTA): clean canal wall and visible tubule openings.

Dentinal surfaces in the four groups that showed different conditioning patterns after push-out tests are described in Figure 3.

## Discussion

The aim of this study was to evaluate whether the irrigating solutions used at the end of the root canal preparation phase and prior to the cementation procedure interfere in the bond strength of fiber posts adhesively luted with self-adhesive resin cement to root dentin. The null hypothesis of this study was rejected, because there was irrigating solution interference on bond strength of fiber posts cemented with self-adhesive cement to root dentin.

The smear layer is an amorphous layer composed of organic and inorganic debris, derived from root canal instrumentation (18). Even resin cement manufacturers do not suggest the use of a specific product prior post cementation, a combination of irrigating solutions is recommended for proper debris removal from the root canal system. Sodium Hypochlorite (NaOCl) and a chelating agent, usually ethylenediaminetetraacetic acid (EDTA), seems to be an appropriate association, as sodium hypochlorite acts selectively to remove organic particles from the smear layer, while EDTA has the ability to remove inorganic material (16,22).

For the effective removal of both inorganic and organic components from the smear layer, it is recommended to treat the dentin surface with 10 mL of 17% EDTA solution, followed by 10 mL of 5% sodium hypochlorite solution (22). Based on this information, the root canal dentin underwent surface treatment in Group 3, suggesting that the association of the two solutions is probably the most suitable for irrigation prior to the cementation procedure when resin luting cements are used (13,16).

In this study, the bond strength values were lower for groups that used isolated irrigant solutions (G2 and G4), and different from Group 3, which combined both solutions (5% NaOCl + 17% EDTA). Sodium hypochlorite, the most commonly used irrigant, can possibly cause root dentin degeneration through organic material removal, especially type I collagen fiber dissolution (23). Based on this evidence, it is possible that bond strength in Group 2 was reduced by the proteolytic action of sodium hypochlorite, which altered or removed organic components from root dentin that can damage the integrity of collagen which is part of the adhesion mechanism (13,24,25).

Another reason for decreased bond strength in Group 2 could be the fact that sodium hypochlorite decomposes into sodium chloride and oxygen in contact with root dentin. Thus, the free oxygen in the dentin surface possibly reduces the bond strength because it inhibits resin cement polymerization and may also generate voids in the cement-dentin interface that could interfere in the resin cement infiltration inside the dentinal tubules as well as in the intertubular dentin (13,26).

In this study, 5% NaOCl used alone caused an adverse effect on bond strength to root dentin similar to that seen in other studies (5,13,24,26,27). Conversely, studies using conventional dual cure resin cements reported an increase in bond strength after treatment with sodium hypochlorite solution, suggesting that NaOCl removes the organic components of dentin, contributing to resin monomer penetration in the structure of demineralized dentin within the dentinal tubules and secondary canals, promoting resin tag formation (28,29).

A probable explanation for the Group 3 bond strength values that were statistically greater than those in Groups 2 and 4, and similar to the control group, is based on the sum of the erosion caused by 17% EDTA, which is a weak acid etching agent with subsequent deproteinization carried out with sodium hypochlorite. Furthermore, this is also associated with the etching of self-adhesive resin cement through its acidic monomers (11). This sum of factors possibly favors the opening of dentinal tubules to facilitate luting agent adhesion to the root dentin (9,30,31).

Group 4 had the lowest bond strength values compared to other groups. Niu et al. (31) reported that when the root canal is irrigated with only 15% EDTA, dentin has smooth and flat appearance and presents regular and separated holes. However, when the canal is irrigated with 15% EDTA solution followed by 6% NaOCl, dentin presents an eroded aspect, with more irregular and rough dentinal tubules, favoring adhesion to root dentin. Perhaps for this reason, Group 3 showed statistically greater bond strength values than Group 4 in this study.

When a self-adhesive cement is used, complete smear layer removal can be a disadvantage for dentin adhesion, because the new conditioning of the dentin surface may be generated by the acidic components of the cement, with negative effect on bond strength (5). This could explain what occurred in Group 4, since the EDTA chelating action removes calcium ions present in the hydroxyapatite crystals; it is also a way of conditioning the root canal, as observed in a study by Radovic *et al.* (11). This would explain the new conditioning, further to the well- known EDTA ability to remove the smear layer (22), which can result in poor adhesion of the self-adhesive cement to dentin in Group 4.

Figure 3 shows SEM microphotographs made for illustrative purposes that demonstrate the following: in G1, a conditioning pattern without significant aperture of dentinal tubules and possible remnants of resinous tags. In G2, presence of the smear layer or some remnants of resin cement covering the surface and dentinal tubules. In G3, a better removal of the smear layer showing open dentinal tubules and possible remnants of resin tags. In G4, a similar smear layer removal pattern with open dentinal tubules and less resin remnants.

Therefore, the null hypothesis was rejected as different irrigant solutions (5% NaOCl and 17% EDTA) interfere with the bond strength of fiber posts cemented with self-adhesive resin cement. However, to confirm the results obtained in this study, new studies can be performed by incorporating other variables.

## Conclusions

Within the limitations of this study, the association of the two irrigating solutions in Group 3 (17% EDTA and 5% NaOCl) provided bond strength statistically similar to Group 1 (control - distilled water), both statistically superior to Group 2 (5% NaOCl) and Group 4 (17% EDTA). Group 4 provided statistically lower bond strength values than the other groups studied.
